# A Multiethnic Replication Study of Plasma Lipoprotein Levels-Associated SNPs Identified in Recent GWAS

**DOI:** 10.1371/journal.pone.0063469

**Published:** 2013-05-22

**Authors:** Emily K. Bryant, Amy S. Dressen, Clareann H. Bunker, John E. Hokanson, Richard F. Hamman, M. Ilyas Kamboh, F. Yesim Demirci

**Affiliations:** 1 Human Genetics, GSPH, University of Pittsburgh, Pittsburgh, Pennsylvania, United States of America; 2 Epidemiology, GSPH, University of Pittsburgh, Pittsburgh, Pennsylvania, United States of America; 3 Epidemiology, Colorado School of Public Health, University of Colorado Denver, Aurora, Colorado, United States of America; National Cancer Institute, National Institutes of Health, United States of America

## Abstract

Genome-wide association studies (GWAS) have identified a number of loci/SNPs associated with plasma total cholesterol (TC), low-density lipoprotein cholesterol (LDL-C), high-density lipoprotein cholesterol (HDL-C), and triglyceride (TG) levels. The purpose of this study was to replicate 40 recent GWAS-identified HDL-C-related new loci in 3 epidemiological samples comprising U.S. non-Hispanic Whites (NHWs), U.S. Hispanics, and African Blacks. In each sample, the association analyses were performed with all 4 major lipid traits regardless of previously reported specific associations with selected SNPs. A total of 22 SNPs showed nominally significant association (*p*<0.05) with at least one lipid trait in at least one ethnic group, although not always with the same lipid traits reported as genome-wide significant in the original GWAS. The total number of significant loci was 10 for TC, 12 for LDL-C, 10 for HDL-C, and 6 for TG levels. Ten SNPs were significantly associated with more than one lipid trait in at least one ethnic group. Six SNPs were significantly associated with at least one lipid trait in more than one ethnic group, although not always with the same trait across various ethnic groups. For 25 SNPs, the associations were replicated with the same genome-wide significant lipid traits in the same direction in at least one ethnic group; at nominal significance for 13 SNPs and with a trend for association for 12 SNPs. However, the associations were not consistently present in all ethnic groups. This observation was consistent with mixed results obtained in other studies that also examined various ethnic groups.

## Introduction

Prior to genome-wide association studies (GWAS), genome-wide linkage scans and candidate gene (positional and/or biological) association studies were the main approaches used to unravel the genetic determinants of complex traits such as plasma lipid/lipoprotein levels. These studies have implicated a number of genes and variants as determinants of plasma total cholesterol (TC), low-density lipoprotein cholesterol (LDL-C), high-density lipoprotein cholesterol (HDL-C) and triglyceride (TG) levels, of which some were more consistently replicated while several others yielded inconsistent results. With the availability of GWAS platforms, it became possible to identify susceptibility variants and genes for complex traits without making *a priori* assumptions.

Several GWAS investigating plasma lipid/lipoprotein traits primarily in subjects of European ancestry have been published to date [1]–[11]. These GWAS confirmed a number of genes previously implicated in influencing the inter-individual variation in four major lipid traits (TC, LDL-C, HDL-C, and TG) in earlier functional and/or candidate gene association studies as well as identified several new loci and genes. A recent meta-analysis of 46 lipid/lipoprotein GWAS comprising of >100,000 individuals, has identified 95 genome-wide significant loci associated with at least one of the four major lipid traits [11].

Only a handful of post-GWAS replication studies published to date have simultaneously examined various (3 or more) ethnic groups, including non-Hispanic Whites (NHWs), Hispanics, and African Americans. Additional independent studies investigating GWAS-identified variants in diverse racial/ethnic groups are needed. In this study, we sought to replicate 40 recent GWAS-identified HDL-C-related loci, which were not among previously established lipid loci/genes, in 3 epidemiological samples comprising of U.S. NHWs, U.S. Hispanics, and African Blacks. In each sample, we performed association analyses with all four major lipid traits (TC, LDL-C, HDL-C, and TG) regardless of previously reported specific associations with selected SNPs.

## Subjects and Methods

### Subjects

The study consisted of 621 NHW and 413 Hispanic non-diabetic subjects drawn from the San Luis Valley Diabetes Study, a population-based case-control study in the San Luis Valley in Southern Colorado, and 787 African Blacks drawn from a study on coronary heart disease (CHD)-related risk factors in Benin City, Nigeria. Detailed information on these studies and subjects can be found elsewhere [12]–[17]. The ages of participants ranged from 24 to 75 in NHWs, 21 to 75 in Hispanics, and 19 to 70 in African Blacks. The demographic and phenotypic characteristics of the study subjects are summarized in **Table 1**. The study was approved by the University of Pittsburgh and University of Colorado Denver Institutional Review Boards and all study participants provided written informed consent.

**Table 1:** Biometric and quantitative data (unadjusted mean ± S.E.) of study samples.

Variable	NHWs (*n = *621)	Hispanics (*n = *413)	African Blacks (*n* = 787)
Gender (Female/Male)	328/293	209/204	292/495
Age (years)	52.82±0.46	51.17±0.62	40.96±0.30
BMI (kg/m^2^)	25.48±0.16	25.76±0.22	22.84±0.14
Total cholesterol (mg/dl)	216.20±1.68	212.79±2.15	171.98±1.39
LDL-C (mg/dl)	138.12±1.53	134.31±1.98	109.23±1.24
HDL-C (mg/dl)	50.79±0.58	48.62±0.65	47.97±0.46
Triglycerides (mg/dl)	139.26±2.80	149.34±3.46	71.80±1.24

### SNP Selection

The purpose of this study was to primarily replicate the HDL-C-related new signals (different from those found in established lipid loci/genes) identified in recent lipid/lipoprotein GWAS. We analyzed a total of 40 SNPs selected from four publications [7]–[9], [11] (**Table 2**). We primarily targeted those SNPs that reached genome-wide level of significance for association with HDL-C levels (n = 36). We also included 4 additional SNPs (shown in *italics* in Table 2) that although did not reach genome-wide level of significance for HDL-C [7], [8], they were either highly significant for HDL-C or modestly significant for HDL-C but genome-wide significant for at least one of the three other lipid traits (TC, LDL-C or TG). Whenever there were more than one significant SNP reported for a given locus by the same group and/or various groups, only one SNP was selected for replication in our samples. Although all these GWAS primarily investigated individuals of European ancestry (EU), one of them [11] also sought replication in various non-European populations, including African Americans. Whenever the information was available, the effects observed in African Americans are shown as ‘concordant’ (AA) or ‘discordant’ (AA*) in Table 2. Of 40 SNPs selected from these GWAS, all were analyzed in our NHW and Hispanic samples whereas only a subset (n = 34) with sufficient minor allele frequency (MAF) was analyzed in our African sample.

**Table 2:** SNPs selected from 4 published GWAS in individuals of European ancestry (EU) for replication in our multiethnic sample§.

SNP	Chr	Gene(s)	Alleles	GWAS Trait(s)	Population	Reference(s)
*rs646776*	1p13	*PSRC1, * ***CELSR2***	A/**G**	TC, LDL-C	EU	Aulchenko et al. 2009 [8]
*rs10889353*	1p31	***DOCK7***	A/**C**	TC, TG	EU	Aulchenko et al. 2009 [8]
*rs10903129*	1p36	***TMEM57***	**G**/A	**TC**	EU	Aulchenko et al. 2009 [8]
rs4660293	1p34	***PABPC4***	A/**G**	HDL-C	EU, AA	Teslovich et al. 2010 [11]
rs1689800	1q25	*LOC100130996, * ***ZNF648***	A/**G**	HDL-C	EU, AA*	Teslovich et al. 2010 [11]
rs2144300	1q42	***GALNT2***	C/**T**	**HDL-C**	EU	Willer et al. 2008 [7]
rs1042034	2p24	***APOB***	T/**C**	**HDL-C**, TG	EU, AA	Teslovich et al. 2010 [11]
rs12328675	2q24	***COBLL1***	T/**C**	**HDL-C**	EU, AA	Teslovich et al. 2010 [11]
rs2972146	2q36	***IRS1***	T/**G**	**HDL-C**, TG	EU, AA	Teslovich et al. 2010 [11]
rs13107325	4q24	***SLC39A8***	C/**T**	HDL-C	EU, AA*	Teslovich et al. 2010 [11]
rs6450176	5q11	***ARL15***	G/**A**	HDL-C	EU, AA	Teslovich et al. 2010 [11]
rs2814944	6p21	***C6orf106***	G/**A**	HDL-C	EU, AA*	Teslovich et al. 2010 [11]
rs605066	6q24	***CITED2***	T/**C**	HDL-C	EU, AA	Teslovich et al. 2010 [11]
rs17145738	7q11	*TBL2, * ***MLXIPL***	C/**T**	**HDL-C**, TG	EU, AA*	Teslovich et al. 2010 [11]
rs4731702	7q32	***KLF14***	C/**T**	**HDL-C**	EU, AA	Teslovich et al. 2010 [11]
rs9987289	8p23	***PPP1R3B***	G/**A**	HDL-C, LDL-C, TC	EU, AA	Teslovich et al. 2010 [11]
rs2293889	8q23	***TRPS1***	G/**T**	HDL-C	EU, AA	Teslovich et al. 2010 [11]
rs471364	9p22	*C9orf52, * ***TTC39B***	T/**C**	HDL-C	EU	Kathiresan et al. 2009 [9]
*rs1323432*	9q31	*PPP3R2, * ***GRIN3A***	**A**/G	HDL-C	EU	Willer et al. 2008 [7]
rs7395662	11p11	*OR4A47, * ***MADD-FOLH1***	**G**/A	HDL-C	EU	Aulchenko et al. 2009 [8]
rs3136441	11p11	*F2, * ***LRP4***	T/**C**	**HDL-C**	EU	Teslovich et al. 2010 [11]
rs2923084	11p15	***AMPD3***	A/**G**	HDL-C	EU, AA	Teslovich et al. 2010 [11]
rs174547	11q12	***FADS1-FADS2-FADS3***	T/**C**	HDL-C, **TG**	EU	Kathiresan et al. 2009 [9]
rs7941030	11q24	*STS-1, * ***UBASH3B***	T/**C**	**HDL-C, TC**	EU, AA	Teslovich et al. 2010 [11]
rs7134375	12p12	***PDE3A***	C/**A**	**HDL-C**	EU, AA	Teslovich et al. 2010 [11]
rs2338104	12q24	*KCTD10, * ***MMAB, MVK***	G/**C**	HDL-C	EU	Kathiresan et al. 2009; Willer et al. 2008 [7], [9]
rs11613352	12q13	***LRP1***	C/**T**	**HDL-C**, TG	EU, AA	Teslovich et al. 2010 [11]
rs2652834	15q22	***LACTB***	G/**A**	HDL-C	EU	Teslovich et al. 2010 [11]
rs2271293	16q22	*NUTF2, * ***CTCF***	**G**/A	HDL-C	EU	Aulchenko et al. 2009 [8]
rs2925979	16q23	***CMIP***	C/**T**	HDL-C	EU, AA	Teslovich et al. 2010 [11]
rs11869286	17q12	***STARD3***	C/**G**	HDL-C	EU, AA	Teslovich et al. 2010 [11]
rs4148008	17q24	***ABCA8***	C/**G**	HDL-C	EU, AA	Teslovich et al. 2010 [11]
rs4129767	17q25	***PGS1***	A/**G**	HDL-C	EU, AA	Teslovich et al. 2010 [11]
rs12967135	18q21	***MC4R***	G/**A**	HDL-C	EU, AA	Teslovich et al. 2010 [11]
rs2967605	19p13	*RAB11B, * ***ANGPTL4***	C/**T**	HDL-C	EU	Kathiresan et al. 2009 [9]
rs737337	19p13	***LOC55908,*** * DOCK6*	T/**C**	HDL-C	EU, AA	Teslovich et al. 2010 [11]
rs386000	19q13	***LILRA3*** *, LILRB2*	G/**C**	**HDL-C**	EU, AA*	Teslovich et al. 2010 [11]
rs1800961	20q13	***HNF4A***	C/**T**	HDL-C, TC	EU, AA*	Kathiresan et al. 2009; Teslovich et al. 2010 [9], [11]
rs6065906	20q13	*FLJ40606, * ***PLTP*** *, PCIF1*	T/**C**	HDL-C, **TG**	EU, AA	Teslovich et al. 2010 [11]
rs181362	22q11	***UBE2L3***	C/**T**	HDL-C	EU, AA	Teslovich et al. 2010 [11]

§ For HDL-C, p-values ranged from 7.7×10^−4^ to 0.02 for 4 SNPs (in *italics*) but were ≤5×10^−8^ for other SNPs as well as for other lipid traits included in the table. When available, replication results in African Africans (AA) are also shown (* = discordant finding with opposite direction of association). Primarily implicated genes, associated alleles, and increased lipid levels are shown in **bold** (decreased levels in unbold). Alleles (on forward or reverse strands) reflect those stated in the original papers.

### Genotyping

DNAs were extracted from either buffy coats (NHWs and Hispanics) or blood clots (African Blacks) using standard methods. Samples were whole-genome amplified using the GenomiPhi DNA Amplification Kit (GE Healthcare Bio-Sciences, Piscataway, NJ) prior to genotyping. Twenty SNPs were genotyped using the TaqMan allelic discrimination method (Applied Biosystems, Foster City, CA) in 621 NHWs, 413 Hispanics, and 787 African Blacks. The other twenty were genotyped using the iPLEX Gold technology (Sequenom, San Diego, CA) in 621 NHWs, 382 Hispanics, and 787 African Blacks. Depending on the genotyping method used, about 7–10% of samples were repeated to test the consistency of genotype calls for each assay.

### Statistical Methods

Concordance of the genotype distribution to Hardy-Weinberg equilibrium (HWE) was tested for each variant using χ2 goodness-of-fit test. Whenever it was necessary to reduce the effects of non-normality, dependent quantitative variables were transformed using either log or square root transformation: ‘log10’ transformation was used for HDL-C and TG levels in NHWs and Hispanics, ‘natural log’ transformation for TC and TG levels in African Blacks, and ‘square root’ transformation for LDL-C and HDL-C levels in African Blacks. Significant covariates for each dependent variable were identified using stepwise regression in order to determine the most parsimonious set of covariates to be included into analysis in each population. Detailed information on the evaluation and effects of covariates in our study samples can be found elsewhere [16]. To test for the effects of genotypes on the means of the quantitative traits, a linear regression analysis was performed (under the additive model) and the results were adjusted for the relevant covariates. For NHWs and Hispanics, the covariates were sex, age, BMI, and smoking. For African Blacks, the covariates were sex, age, waist measurement, exercise (minutes walking or bicycling to work each day), and staff level (junior or senior). The R statistical software package (version 2.12.2, http://www.r-project.org) was used to perform all analyses. Because this was a replication study, we considered a nominal *p*<0.05 as evidence of association. In addition, because we compared our results to those obtained in large GWAS and meta-analyses that included several thousand subjects, we have also discussed the results of the SNPs that showed a trend (p-values between 0.05–0.20) for ‘the same direction’ of association with ‘the same genome-wide significant lipid trait’ reported in the original GWAS.

## Results

The genotype call rates were very high (≥95%) for almost all assays and only a small number of SNPs showed lower call rates: 1 in NHWs (87%), 3 in Hispanics (between 92–95%), and 2 in African Blacks (between 92–95%). No SNP showed low call rates across all populations genotyped. Discrepancy among replicates was detected for only one assay (rs174547) for which the discrepancy rate was 0.5%.

The association results for 4 major lipid traits examined in 3 ethnic groups are summarized in **Table 3**. Most SNPs differed in allele frequencies among various ethnic groups. For 10 SNPs (shown in *italics* in Table 3), it was not always the same allele that was the minor allele across various ethnic groups and this was taken into account when making cross-sample comparison because the genotypic effects were modeled as the additive effect of the population-specific minor allele in each ethnic group.

**Table 3:** Summary of SNP associations with 4 lipid traits in our multi-ethnic study samples§.

Chr Gene(s)	SNP Traits	NHWs			Hispanics			African Blacks		
		MAF	Association		MAF	Association		MAF	Association	
**1p13**	**rs646776**	**C - 0.212**	**beta**	**p-value**	**C - 0.258**	**beta**	**p-value**	**C - 0.348**	**beta**	**p-value**
*PSRC1*	TC		−6.801	**0.012**		1.515	0.675		0.006	0.616
*CELSR2*	LDL-C		−5.754	**0.023**		0.712	0.830		−0.044	0.601
	HDL-C		0.003	0.711		0.012	0.163		0.062	0.199
	TG		−0.005	0.722		−0.019	0.218		0.004	0.857
**1p31**	**rs10889353**	**C - 0.337**	**beta**	**p-value**	**C - 0.365**	**beta**	**p-value**	**C - 0.447**	**beta**	**p-value**
*DOCK7*	TC		−5.183	**0.031**		−4.087	0.190		−0.003	0.790
	LDL-C		−2.223	0.320		−2.515	0.382		−0.004	0.964
	HDL-C		−9.2×10^−5^	0.988		0.008	0.305		−0.029	0.546
	TG		−0.046	**4.9×10** ^−**5**^		−0.038	**0.004**		−0.028	0.172
**1p36**	**rs10903129**	**A - 0.439**	**beta**	**p-value**	**A - 0.492**	**beta**	**p-value**	**A - 0.222**	**beta**	**p-value**
*TMEM57*	TC		1.767	0.448		0.283	0.926		0.002	0.897
	LDL-C		1.680	0.437		−0.553	0.842		0.048	0.621
	HDL-C		−0.008	0.186		0.011	0.110		0.014	0.800
	TG		0.018	0.099		−0.006	0.669		0.005	0.829
**1p34**	**rs4660293**	**G - 0.212**	**beta**	**p-value**	**G - 0.206**	**beta**	**p-value**		N.A^*^	
*PABPC4*	TC		0.796	0.765		1.972	0.630			
	LDL-C		0.673	0.785		3.258	0.386			
	HDL-C		3.3×10^−4^	0.961		−0.010	0.278			
	TG		0.002	0.902		−0.002	0.917			
**1q25**	**rs1689800**	**G - 0.347**	**beta**	**p-value**	**G - 0.346**	**beta**	**p-value**	**G - 0.264**	**beta**	**p-value**
*ZNF648*	TC		−2.747	0.268		−0.820	0.798		−0.017	0.170
	LDL-C		−2.481	0.279		−0.014	0.996		−0.141	0.117
	HDL-C		−0.010	0.121		−0.007	0.378		0.019	0.713
	TG		0.013	0.261		−0.001	0.945		0.009	0.689
**1q42**	**rs2144300**	**C - 0.384**	**beta**	**p-value**	**C - 0.425**	**beta**	**p-value**	***T - 0.040***	***beta***	***p-value***
*GALNT2*	TC		4.585	0.051		1.537	0.631		*0.039*	*0.172*
	LDL-C		4.498	**0.040**		0.636	0.830		*0.233*	*0.260*
	HDL-C		−0.011	0.060		−0.005	0.534		*0.100*	*0.401*
	TG		0.030	**0.006**		0.009	0.533		−*0.001*	*0.990*
**2p24**	**rs1042034**	**G - 0.215**	**beta**	**p-value**	**G - 0.298**	**beta**	**p-value**	**G - 0.119**	**beta**	**p-value**
*APOB*	TC		−2.519	0.361		−12.814	**2.0×10** ^−**4**^		−0.012	0.504
	LDL-C		−2.721	0.285		−12.508	**8.0×10** ^−**5**^		−0.102	0.432
	HDL-C		0.006	0.398		−0.005	0.528		0.078	0.300
	TG		−0.019	0.142		−0.006	0.669		−0.050	0.118
**2q24**	**rs12328675**	**C - 0.143**	**beta**	**p-value**	**C - 0.101**	**beta**	**p-value**	**C - 0.201**	**beta**	**p-value**
*COBLL1*	TC		−3.158	0.315		4.136	0.425		−0.016	0.260
	LDL-C		−4.021	0.169		1.913	0.686		−0.160	0.119
	HDL-C		0.002	0.814		0.007	0.559		−0.002	0.968
	TG		0.009	0.522		0.023	0.289		0.022	0.383
**2q36**	**rs2972146**	**C - 0.377**	**beta**	**p-value**	**C - 0.230**	**beta**	**p-value**	**C - 0.123**	**beta**	**p-value**
*IRS1*	TC		0.726	0.756		−1.986	0.561		0.006	0.733
	LDL-C		1.337	0.536		−0.623	0.842		0.019	0.879
	HDL-C		0.005	0.373		−0.005	0.562		−0.035	0.622
	TG		−0.004	0.710		−0.023	0.111		0.027	0.361
**4q24**	**rs13107325**	**T - 0.068**	**beta**	**p-value**	**T - 0.045**	**beta**	**p-value**		N.A^*^	
*SLC39A8*	TC		−1.391	0.762		−3.310	0.667			
	LDL-C		−3.258	0.444		0.236	0.973			
	HDL-C		0.022	0.064		−0.032	0.066			
	TG		−0.013	0.554		0.008	0.812			
**5q11**	**rs6450176**	**A - 0.240**	**beta**	**p-value**	**A - 0.296**	**beta**	**p-value**	**A - 0.317**	**beta**	**p-value**
*ARL15*	TC		1.573	0.552		−3.270	0.337		−0.003	0.832
	LDL-C		1.193	0.627		−2.020	0.517		0.004	0.967
	HDL-C		0.003	0.668		−0.005	0.514		−0.040	0.425
	TG		−0.002	0.859		−0.006	0.662		0.003	0.906
**6p21**	**rs2814944**	**A - 0.163**	**beta**	**p-value**	**A - 0.132**	**beta**	**p-value**	**A - 0.342**	**beta**	**p-value**
*C6orf106*	TC		−1.491	0.623		−6.484	0.156		0.005	0.681
	LDL-C		−0.349	0.901		−7.810	0.062		0.055	0.527
	HDL-C		−0.007	0.377		−0.008	0.428		−0.057	0.250
	TG		−0.002	0.879		0.026	0.181		0.027	0.202
**6q24**	**rs605066**	**C - 0.403**	**beta**	**p-value**	**C - 0.406**	**beta**	**p-value**	***T - 0.402***	***beta***	***p-value***
*CITED2*	TC		−4.002	0.092		1.230	0.699		*0.013*	*0.248*
	LDL-C		−4.946	**0.025**		1.408	0.628		*0.105*	*0.208*
	HDL-C		0.003	0.675		−0.010	0.170		*0.014*	*0.776*
	TG		0.001	0.918		0.015	0.266		*0.001*	*0.969*
**7q11**	**rs17145738**	**T - 0.108**	**beta**	**p-value**	**T - 0.071**	**beta**	**p-value**	**T - 0.086**	**beta**	**p-value**
*TBL2*	TC		2.133	0.558		1.020	0.869		−0.001	0.962
*MLXIPL*	LDL-C		4.142	0.219		2.486	0.663		0.075	0.611
	HDL-C		0.011	0.219		−0.001	0.940		−0.044	0.605
	TG		−0.041	**0.016**		−0.020	0.448		0.014	0.697
**7q32**	**rs4731702**	**T - 0.494**	**beta**	**p-value**	**T - 0.436**	**beta**	**p-value**	**T - 0.184**	**beta**	**p-value**
*KLF14*	TC		0.120	0.960		0.638	0.845		−0.011	0.425
	LDL-C		−0.881	0.689		1.459	0.625		−0.126	0.212
	HDL-C		0.018	**0.003**		−0.009	0.222		0.012	0.840
	TG		−0.012	0.302		0.007	0.617		0.012	0.620
**8p23**	**rs9987289**	**A - 0.091**	**beta**	**p-value**	**A - 0.178**	**beta**	**p-value**	**A - 0.191**	**beta**	**p-value**
*PPP1R3B*	TC		0.971	0.808		−7.966	0.051		−0.038	**0.009**
	LDL-C		1.565	0.671		−6.099	0.106		−0.273	**0.010**
	HDL-C		0.002	0.867		−0.025	**0.011**		−0.065	0.276
	TG		0.006	0.757		0.017	0.340		0.035	0.177
**8q23**	**rs2293889**	**T - 0.442**	**beta**	**p-value**	**T - 0.445**	**beta**	**p-value**	**T - 0.040**	**beta**	**p-value**
*TRPS1*	TC		−0.410	0.861		1.852	0.551		−0.012	0.680
	LDL-C		0.715	0.743		2.085	0.467		0.014	0.947
	HDL-C		−0.005	0.399		0.005	0.506		−0.168	0.148
	TG		−0.013	0.258		0.001	0.914		−0.002	0.969
**9p22**	**rs471364**	**C - 0.125**	**beta**	**p-value**	**C - 0.105**	**beta**	**p-value**	**C - 0.206**	**beta**	**p-value**
*TTC39B*	TC		−2.177	0.532		−6.466	0.233		−0.009	0.524
	LDL-C		2.511	0.437		−3.277	0.515		−0.060	0.551
	HDL-C		−0.016	0.063		−0.015	0.253		0.014	0.813
	TG		−0.016	0.344		−0.007	0.776		−0.002	0.948
**9q31**	**rs1323432**	**G - 0.115**	**beta**	**p-value**	**G - 0.087**	**beta**	**p-value**		N.A^*^	
*PPP3R2*	TC		11.295	**0.002**		7.703	0.148			
*GRIN3A*	LDL-C		8.951	**0.009**		4.455	0.364			
	HDL-C		−0.003	0.754		0.027	**0.038**			
	TG		0.026	0.141		−0.006	0.799			
**11p11**	**rs7395662**	**A - 0.373**	**beta**	**p-value**	**A - 0.323**	**beta**	**p-value**	***G - 0.407***	***beta***	***p-value***
*OR4A47*	TC		2.869	0.225		−8.075	**0.014**		−*0.002*	*0.848*
*MADD-*	LDL-C		3.129	0.154		−7.407	**0.014**		−*0.011*	*0.898*
*FOLH1*	HDL-C		−2.9×10^−6^	1.000		0.008	0.289		*0.025*	*0.608*
	TG		0.008	0.484		−0.018	0.204		−*0.016*	*0.448*
**11p11**	**rs3136441**	**C - 0.137**	**beta**	**p-value**	**C - 0.268**	**beta**	**p-value**		N.A^*^	
*F2*	TC		−0.523	0.874		−4.952	0.168			
*LRP4*	LDL-C		−0.607	0.843		−4.207	0.202			
	HDL-C		0.006	0.469		−0.006	0.444			
	TG		0.013	0.420		−9.8E-04	0.950			
**11p15**	**rs2923084**	**G - 0.179**	**beta**	**p-value**	**G - 0.307**	**beta**	**p-value**	***A - 0.474***	***beta***	***p-value***
*AMPD3*	TC		2.969	0.307		−3.556	0.289		*0.006*	*0.578*
	LDL-C		3.145	0.243		−2.051	0.508		*0.032*	*0.693*
	HDL-C		−0.015	**0.048**		−0.004	0.591		*0.019*	*0.688*
	TG		0.020	0.135		−0.002	0.870		*0.006*	*0.744*
**11q12**	**rs174547^‡^**	**C - 0.345**	**beta**	**p-value**	***T - 0.441***	***beta***	***p-value***		N.A^*^	
*FADS1*	TC		−3.992	0.112		*5.627*	*0.072*			
*FADS2*	LDL-C		−2.996	0.196		*5.716*	***0.048***			
*FADS3*	HDL-C		−0.010	0.098		*0.001*	*0.901*			
	TG		−0.011	0.371		−*0.005*	*0.711*			
**11q24**	**rs7941030**	**C - 0.367**	**beta**	**p-value**	**C - 0.294**	**beta**	**p-value**	**C - 0.444**	**beta**	**p-value**
*UBASH3B*	TC		5.359	**0.030**		−0.860	0.797		−0.011	0.315
	LDL-C		3.490	0.131		−0.729	0.814		−0.084	0.302
	HDL-C		0.005	0.410		−0.005	0.511		0.038	0.412
	TG		0.021	0.067		0.015	0.292		−0.040	**0.043**
**12p12**	**rs7134375**	**A - 0.458**	**beta**	**p-value**	***C - 0.497***	***beta***	***p-value***	**A - 0.308**	**beta**	**p-value**
*PDE3A*	TC		1.439	0.525		−*3.594*	*0.269*		0.006	0.611
	LDL-C		0.531	0.799		−*3.243*	*0.278*		0.013	0.885
	HDL-C		0.011	**0.049**		*0.003*	*0.695*		0.059	0.240
	TG		−0.009	0.412		−*0.006*	*0.687*		−0.002	0.910
**12q24**	**rs2338104**	**C - 0.429**	**beta**	**p-value**	**C - 0.486**	**beta**	**p-value**	**C - 0.196**	**beta**	**p-value**
*KCTD10*	TC		0.357	0.877		−5.109	0.098		−0.010	0.477
*MMAB*	LDL-C		0.705	0.740		−5.855	**0.041**		0.089	0.390
*MVK*	HDL-C		−0.005	0.383		0.002	0.777		−0.187	**0.002**
	TG		0.006	0.601		−0.004	0.751		−0.043	0.094
**12q13**	**rs11613352**	**T - 0.245**	**beta**	**p-value**	**T - 0.382**	**beta**	**p-value**	**T - 0.063**	**beta**	**p-value**
*LRP1*	TC		1.291	0.635		1.589	0.621		−0.008	0.713
	LDL-C		0.244	0.923		1.545	0.599		−0.250	0.132
	HDL-C		−0.002	0.722		−0.004	0.569		0.198	**0.035**
	TG		−0.003	0.815		0.006	0.669		0.054	0.183
**15q22**	**rs2652834**	**T - 0.188**	**beta**	**p-value**	**T - 0.154**	**beta**	**p-value**	**T - 0.338**	**beta**	**p-value**
*LACTB*	TC		−0.576	0.847		−2.073	0.638		0.009	0.429
	LDL-C		−0.532	0.848		−3.853	0.340		0.055	0.522
	HDL-C		−0.001	0.918		−0.009	0.368		0.013	0.792
	TG		0.006	0.682		0.018	0.341		0.001	0.945
**16q22**	**rs2271293**	**A - 0.121**	**beta**	**p-value**	**A - 0.155**	**beta**	**p-value**	**A - 0.081**	**beta**	**p-value**
*NUTF2*	TC		7.170	**0.044**		2.092	0.626		0.010	0.635
	LDL-C		4.285	0.192		3.055	0.440		0.126	0.387
	HDL-C		0.023	**0.009**		0.006	0.549		0.090	0.284
	TG		−0.003	0.843		−0.014	0.427		−0.056	0.120
**16q23**	**rs2925979**	**A - 0.287**	**beta**	**p-value**	**A - 0.203**	**beta**	**p-value**	**A - 0.293**	**beta**	**p-value**
*CMIP*	TC		1.514	0.544		−4.750	0.224		−0.032	**0.008**
	LDL-C		3.074	0.184		−3.317	0.355		−0.203	**0.022**
	HDL-C		−0.007	0.273		−0.008	0.383		−0.053	0.290
	TG		−0.014	0.236		−0.007	0.687		−0.016	0.459
**17q12**	**rs11869286**	**G - 0.357**	**beta**	**p-value**	**G - 0.387**	**beta**	**p-value**	***C - 0.172***	***beta***	***p-value***
*STARD3*	TC		3.194	0.182		0.465	0.885		−*0.022*	*0.128*
	LDL-C		2.940	0.185		0.435	0.883		−*0.191*	*0.069*
	HDL-C		−0.006	0.286		−0.003	0.732		−*0.106*	*0.077*
	TG		0.009	0.431		0.003	0.845		*0.034*	*0.182*
**17q24**	**rs4148008**	**G - 0.332**	**beta**	**p-value**	**G - 0.272**	**beta**	**p-value**	***C - 0.409***	***beta***	***p-value***
*ABCA8*	TC		1.800	0.458		3.567	0.323		−*0.007*	*0.541*
	LDL-C		2.287	0.310		3.112	0.351		−*0.007*	*0.938*
	HDL-C		3.4×10^−4^	0.957		−0.003	0.694		−*0.008*	*0.874*
	TG		0.008	0.488		0.010	0.539		*0.005*	*0.817*
**17q25**	**rs4129767**	**A - 0.498**	**beta**	**p-value**	***G - 0.461***	***beta***	***p-value***	**A - 0.313**	**beta**	**p-value**
*PGS1*	TC		3.112	0.183		*6.496*	***0.038***		0.027	**0.026**
	LDL-C		3.988	0.065		*6.770*	***0.019***		0.135	0.120
	HDL-C		−0.014	**0.015**		−*0.007*	*0.385*		0.064	0.205
	TG		0.027	**0.013**		*0.015*	*0.269*		0.009	0.685
**18q21**	**rs12967135**	**A - 0.235**	**beta**	**p-value**	**A - 0.175**	**beta**	**p-value**	**A - 0.324**	**beta**	**p-value**
*MC4R*	TC		−4.651	0.078		6.168	0.121		0.018	0.163
	LDL-C		−3.709	0.130		6.215	0.090		0.127	0.166
	HDL-C		−0.001	0.848		−0.010	0.295		0.126	**0.017**
	TG		−0.018	0.152		0.004	0.824		−0.029	0.206
**19p13**	**rs2967605**	**T - 0.169**	**beta**	**p-value**	**T - 0.214**	**beta**	**p-value**	**T - 0.237**	**beta**	**p-value**
*RAB11B*	TC		1.575	0.598		−1.284	0.734		0.013	0.324
*ANGPTL4*	LDL-C		1.256	0.649		0.781	0.823		0.123	0.199
	HDL-C		−0.003	0.678		−0.012	0.189		−0.003	0.959
	TG		0.007	0.625		−0.014	0.377		−0.007	0.780
**19p13**	**rs737337**	**C - 0.079**	**beta**	**p-value**	**C - 0.267**	**beta**	**p-value**	***T - 0.483***	***beta***	***p-value***
*DOCK6*	TC		−2.436	0.555		−4.401	0.195		*0.012*	*0.284*
	LDL-C		−4.911	0.198		−6.843	**0.029**		*0.050*	*0.536*
	HDL-C		0.014	0.180		−0.003	0.723		*0.048*	*0.300*
	TG		0.012	0.527		0.018	0.220		*0.003*	*0.881*
**19q13**	**rs386000**	**G - 0.203**	**beta**	**p-value**	**G - 0.437**	**beta**	**p-value**	**G - 0.183**	**beta**	**p-value**
*LILRA3*	TC		1.725	0.524		4.067	0.224		0.003	0.857
*LILRB2*	LDL-C		3.353	0.180		2.619	0.396		−0.025	0.810
	HDL-C		−0.003	0.629		1.2E-04	0.988		0.082	0.169
	TG		−0.005	0.703		0.020	0.175		−0.027	0.292
**20q13**	**rs1800961**	**T - 0.032**	**beta**	**p-value**	**T - 0.033**	**beta**	**p-value**		N.A^*^	
*HNF4A*	TC		−0.463	0.943		−11.381	0.169			
	LDL-C		1.910	0.748		−9.706	0.211			
	HDL-C		−0.009	0.568		0.005	0.794			
	TG		−0.028	0.345		−0.042	0.238			
**20q13**	**rs6065906**	**C - 0.173**	**beta**	**p-value**	**C - 0.097**	**beta**	**p-value**	**C - 0.158**	**beta**	**p-value**
*PLTP*	TC		3.707	0.213		2.927	0.583		−0.012	0.432
*PCIF1*	LDL-C		2.263	0.416		1.173	0.811		−0.088	0.424
	HDL-C		−0.001	0.921		−0.018	0.138		−0.060	0.336
	TG		0.010	0.461		0.046	**0.044**		0.019	0.487
**22q11**	**rs181362**	**T - 0.201**	**beta**	**p-value**	**T - 0.366**	**beta**	**p-value**	**T - 0.445**	**beta**	**p-value**
*UBE2L3*	TC		0.284	0.921		1.535	0.625		0.005	0.673
	LDL-C		0.948	0.721		1.257	0.664		0.074	0.365
	HDL-C		−0.002	0.790		0.003	0.684		−0.017	0.710
	TG		−0.002	0.896		0.002	0.878		0.012	0.547

§ Significant p-values (<0.05) are shown in **bold**. ‘Log10’ transformation was used for HDL-C and TG levels in Non-Hispanic Whites (NHWs) and Hispanics, ‘natural log’ transformation for TC and TG levels in African Blacks, and ‘square root’ transformation for LDL-C and HDL-C levels in African Blacks. The genotypic effects were modeled as the additive effect of the population-specific minor allele in each ethnic group (minor alleles that differed from those in NHWs are shown in *italics*). The results were adjusted for relevant covariates in each ethnic group. ^*^N.A: These SNPs were not analyzed in African Blacks among which they did not have sufficient minor allele frequency (MAF). Six SNPs showed lower genotyping call rate (<95%) in one of the 3 ethnic groups studied (MAF underlined) while the remaining SNPs had high call rates in all ethnic groups. **^‡^**This SNP showed low rate (0.5%) of discrepancy among replicates included in genotyping.

A total of 22 SNPs showed nominally significant association (*p*<0.05, shown in **bold** in Table 3) with at least one lipid trait in at least one ethnic group with a total of 40 significant p-values, although not always with the same lipid traits reported as genome-wide significant in the original GWAS (13 of 22 significant SNPs showed replicated association with the same lipid traits reported in the original GWAS). The total number of significant loci was 10 for TC, 12 for LDL-C, 10 for HDL-C, and 6 for TG levels. Ten SNPs were significantly associated with more than one lipid trait in at least one ethnic group and these associations were as follows: 6 SNPs (*CELSR2*/rs646776, *APOB*/rs1042034, *PPP1R3B*/rs9987289, *GRIN3A*/rs1323432, *OR4A47*/rs7395662, *CMIP*/rs2925979) with TC and LDL-C, *DOCK7*/rs10889353 with TC and TG, *GALNT2*/rs2144300 with LDL-C and TG, *NUTF2*/rs2271293 with TC and HDL-C, and *PGS1*/rs4129767 with TC, LDL-C, HDL-C, and TG. Six SNPs (*DOCK7*/rs10889353, *PPP1R3B*/rs9987289, *GRIN3A*/rs1323432, *UBASH3B*/rs7941030, *MMAB-MVK*/rs2338104, *PGS1*/rs4129767) were significantly associated with at least one lipid trait in more than one ethnic group, although not always with the same trait across various ethnic groups.

In NHWs, 12 SNPs showed significant association with at least one lipid trait. The most significant SNP for each trait was: *GRIN3A*/rs1323432 with TC (*p* = 0.002) and LDL-C (*p* = 0.009), *KLF14*/rs4731702 with HDL-C (*p* = 0.003), and *DOCK7*/rs10889353 with TG (*p* = 4.9×10^−5^). Six SNPs (*CELSR2*/rs646776, *DOCK7*/rs10889353, *GALNT2*/rs2144300, *GRIN3A*/rs1323432, *NUTF2*/rs2271293, and *PGS1*/rs4129767) were significantly associated with more than one lipid trait.

In Hispanics, 10 SNPs showed significant association with at least one lipid trait. The most significant SNP for each trait was: *APOB*/rs1042034 with TC (*p* = 2.0×10^−4^) and LDL-C (*p* = 8.0×10^−5^), *PPP1R3B*/rs9987289 with HDL-C (*p* = 0.011), and *DOCK7*/rs10889353 with TG (*p* = 0.004). Three SNPs (*APOB*/rs1042034, *OR4A47*/rs7395662, and *PGS1*/rs4129767) were significantly associated with more than one lipid trait.

In African Blacks, 7 SNPs showed significant association with at least one lipid trait. The most significant SNP for each trait was: *CMIP*/rs2925979 with TC (*p* = 0.008), *PPP1R3B*/rs9987289 with LDL-C (*p* = 0.010), *MMAB-MVK*/rs2338104 with HDL-C (*p* = 0.002), and *UBASH3B*/rs7941030 with TG (*p* = 0.043). Two SNPs (*PPP1R3B*/rs9987289, *CMIP*/rs2925979) were significantly associated with more than one lipid trait.

For 25 of 40 SNPs analyzed (34 in African Blacks), we were able to replicate the GWAS associations (with the same lipid trait in the same direction) in at least one ethnic group that we studied; at nominal significance (*p*<0.05) for 13 SNPs and with a trend for association (p-values between 0.05–0.20) for 12 SNPs (please see **Table 4** for details). There were additional SNPs with higher p-values that showed similar trends for effects on the same lipid traits as seen in the original GWAS. Of 6 SNPs showing genome-wide significance for TC levels, we were able to replicate the associations in the same direction for 5 SNPs (at nominal significance for 4 SNPs and with a trend for association for one SNP) in at least one ethnic group studied. Of 2 SNPs showing genome-wide significance for LDL-C levels, we were able to replicate the associations in the same direction for both SNPs at nominal significance in at least one ethnic group studied. Of 36 SNPs with genome-wide significance and one with *p* = 7.7×10^−4^ for HDL-C levels, we were able to replicate the associations in the same direction for 18 SNPs (at nominal significance for 8 SNPs and with a trend for association for 10 SNPs) in at least one ethnic group studied. Two SNPs (*PGS1*/rs4129767 and *MC4R*/12967135) showed significant but discordant results (opposite direction) for association with HDL-C levels as compared to the original GWAS. Of 7 SNPs showing genome-wide significance for TG levels, we were able to replicate the associations in the same direction for 5 SNPs (at nominal significance for 3 SNPs and with a trend for association for 2 SNPs) in at least one ethnic group studied.

**Table 4:** SNPs significantly (*p*<0.05) associated with at least one lipid trait in at least one ethnic group in our study, as well as those that showed a trend for the same direction of association (*p* between 0.05–0.20, *italic traits*) as seen for at least one genome-wide significant lipid trait in the original GWAS§ (only relevant observations have been included in the table).

Locus	SNP	NHWs MAF	HSPsMAF	ABs MAF	NHWs Traits	HSPs Traits	ABsTraits	GWAS Alleles	GWAS Trait(s)	Population
**1p13**	**rs646776**	C-0.212	C-0.258	C-0.348	TC, LDL-C	**–**	**–**	A/**G**	TC, LDL-C	EU
**1p31**	**rs10889353**	C-0.337	C-0.365	C-0.447	TC, TG	TG**,** *TC*	*TG*	A/**C**	TC, TG	EU
**1q25**	**rs1689800**	G-0.347	G-0.346	G-0.264	*HDL-C*	**–**	**–**	A/**G**	HDL-C	EU, AA*
**1q42**	**rs2144300**	*C-0.384*	*C-0.425*	T-0.040	**LDL-C,** *HDL-C* **, TG**	**–**	**–**	C/**T**	**HDL-C**	EU
**2p24**	**rs1042034**	G-0.215	G-0.298	G-0.119	*TG*	TC, LDL-C	*TG*	T/**C**	**HDL-C**, TG	EU, AA
**2q36**	**rs2972146**	C-0.377	C-0.230	C-0.123	**–**	*TG*	**–**	T/**G**	**HDL-C**, TG	EU, AA
**4q24**	**rs13107325**	T-0.068	T-0.045	na	**–**	*HDL-C*	na	C/**T**	HDL-C	EU, AA*
**6q24**	**rs605066**	C-0.403	C-0.406	*T-0.402*	LDL-C	*HDL-C*	**–**	T/**C**	HDL-C	EU, AA
**7q11**	**rs17145738**	T-0.108	T-0.071	T-0.086	TG	**–**	**–**	C/**T**	**HDL-C**, TG	EU, AA*
**7q32**	**rs4731702**	T-0.494	T-0.436	T-0.184	**HDL-C**	**–**	**–**	C/**T**	**HDL-C**	EU, AA
**8p23**	**rs9987289**	A-0.091	A-0.178	A-0.191	**–**	*TC*, HDL-C, *LDL-C*	TC, LDL-C	G/**A**	HDL-C, LDL-C, TC	EU, AA
**8q23**	**rs2293889**	T-0.442	T-0.445	T-0.040	**–**	**–**	*HDL-C*	G/**T**	HDL-C	EU, AA
**9p22**	**rs471364**	C-0.125	C-0.105	C-0.206	*HDL-C*	**–**	**–**	T/**C**	HDL-C	EU
**9q31**	**rs1323432**	*G-0.115*	*G-0.087*	na	**TC, LDL-C**	**HDL-C**	na	**A**/G	HDL-C	EU
**11p11**	**rs7395662**	*A-0.373*	*A-0.323*	G-0.407	**–**	TC, LDL-C	**–**	**G**/A	HDL-C	EU
**11p15**	**rs2923084**	G-0.179	G-0.307	*A-0.474*	HDL-C	**–**	**–**	A/**G**	HDL-C	EU, AA
**11q12**	**rs174547**	C-0.345	*T-0.441*	na	*HDL-C*	**LDL-C**	na	T/**C**	HDL-C, **TG**	EU
**11q24**	**rs7941030**	C-0.367	C-0.294	C-0.444	**TC**	**–**	TG	T/**C**	**HDL-C**, **TC**	EU, AA
**12p12**	**rs7134375**	A-0.458	*C-0.497*	A-0.308	**HDL-C**	**–**	**–**	C/**A**	**HDL-C**	EU, AA
**12q24**	**rs2338104**	C-0.429	C-0.486	C-0.196	**–**	LDL-C	HDL-C	G/**C**	HDL-C	EU
**12q13**	**rs11613352**	T-0.245	T-0.382	T-0.063	**–**	**–**	**HDL-C**	C/**T**	**HDL-C**, TG	EU, AA
**16q22**	**rs2271293**	*A-0.121*	*A-0.155*	*A-0.081*	**TC, HDL-C**	**–**	**–**	**G**/A	HDL-C	EU
**16q23**	**rs2925979**	A-0.287	A-0.203	A-0.293	**–**	**–**	TC, LDL-C	C/**T**	HDL-C	EU, AA
**17q25**	**rs4129767**	*A-0.498*	G-0.461	*A-0.313*	HDL-C**, TG**	**TC, LDL-C**	**TC**	A/**G**	HDL-C	EU, AA
**18q21**	**rs12967135**	A-0.235	A-0.175	A-0.324	**–**	**–**	**HDL-C**	G/**A**	HDL-C	EU, AA
**19p13**	**rs2967605**	T-0.169	T-0.214	T-0.237	**–**	*HDL-C*	**–**	C/**T**	HDL-C	EU
**19p13**	**rs737337**	C-0.079	C-0.267	*T-0.483*	**–**	LDL-C	**–**	T/**C**	HDL-C	EU, AA
**19q13**	**rs386000**	G-0.203	G-0.437	G-0.183	**–**	**–**	***HDL-C***	G/**C**	**HDL-C**	EU, AA*
**20q13**	**rs1800961**	T-0.032	T-0.033	na	**–**	*TC*	na	C/**T**	HDL-C, TC	EU, AA*
**20q13**	**rs6065906**	C-0.173	C-0.097	C-0.158	**–**	**TG,** *HDL-C*	**–**	T/**C**	HDL-C, **TG**	EU, AA

§ Alternate alleles evaluated as compared to GWAS alleles (in **bold**), for which opposite effects are expected, are shown in *italics*. Up-regulated lipid traits are shown in **bold** vs. down-regulated in unbold. MAF: Minor allele frequency, NHWs: Non-Hispanic Whites, HSPs: Hispanics, ABs: African Blacks, EU: Individuals of European descent included in original GWAS, AA: African American replication sample in original GWAS (* = discordant finding with opposite direction of association), na: not analyzed.

For 12 SNPs, we observed significant associations with lipid traits other than those reported as genome-wide significant in the original GWAS (Table 4). Three of these 12 SNPs did not show any significant association or a trend for the same direction association with the traits identified as genome-wide significant in the original GWAS.

## Discussion

We conducted a replication study on 40 recent GWAS-identified new loci that were associated with HDL-C levels [7]–[9], [11] using a multiethnic sample comprising of 3 different ethnic groups (NHWs, Hispanics, and African Blacks). Since MAFs of 6 of 40 SNPs were low in African Blacks, only 34 SNPs were included in the analysis in this group. Although we primarily focused on GWAS signals influencing plasma HDL-C levels (with or without effects on other lipids) when selecting the SNPs to be replicated in our study, we performed association analyses with all four major lipid traits (TC, LDL-C, HDL-C, and TG) regardless of previously reported specific associations with selected SNPs.

Given that our study examined various ethnic groups and our sample sizes were modest as compared to those used in large GWAS and meta-analyses (which examined several thousand subjects), we have used nominal significance (*p*<0.05) for replication and also taken into account the p-values between 0.05 and 0.20 (whenever there was a trend for the same direction of association with the same genome-wide significant lipid trait) when comparing our results with the original GWAS signals. The use of nominal significance for replication and for generalization to non-European populations has been widely employed and applied by some large consortiums [18] and also an acceptable criterion for publications [19]. The comparison of our results to those in original GWAS from which the SNPs were selected has been summarized in Table 4, by including all SNPs that were significantly (*p*<0.05) associated with at least one lipid trait in at least one ethnic group in our study as well as the SNPs that showed a trend for the same direction of association as seen for at least one genome-wide significant (*p*≤5×10^−8^) lipid trait in the original GWAS.

Our analysis revealed a total of 22 SNPs/loci with nominally significant association (*p*<0.05) with at least one lipid trait in at least one ethnic group, although not always with the same lipid traits reported as genome-wide significant in the original GWAS (13 of 22 significant SNPs showed replicated association with originally reported lipid traits in GWAS). Although our SNP selection was biased toward including primarily HDL-C-related variants, we identified a similar number of significant associations with TC (n = 10), LDL-C (n = 12), and HDL-C (n = 10), but relatively less with TG (n = 6). This observation suggests that replication studies should not restrict their analyses of GWAS signals to only specific lipid traits that were initially reported but should rather evaluate all lipid traits for a given variant. Ten loci (*CELSR2*, *APOB*, *PPP1R3B*, *GRIN3A*, *OR4A47*, *CMIP*, *DOCK7*, *GALNT2*, *NUTF2*, and *PGS1*) were significantly associated with more than one lipid trait in at least one ethnic group studied. Six loci (*DOCK7*, *PPP1R3B*, *GRIN3A*, *UBASH3B*, *MMAB-MVK*, *PGS1*) were significantly associated with at least one lipid trait in more than one ethnic group, although not always with the same trait across various ethnic groups.

In NHWs, 12 SNPs showed nominally significant association (*p*<0.05) with at least one lipid trait and 6 were significantly associated with more than one lipid trait. These numbers were 10 and 3 in Hispanics and 7 and 2 in African Blacks. The most significant SNPs for TC levels were *GRIN3A*/rs1323432 in NHWs, *APOB*/rs1042034 in Hispanics, and *CMIP*/rs2925979 in African Blacks. The most significant SNPs for LDL-C levels were *GRIN3A*/rs1323432 in NHWs, *APOB*/rs1042034 in Hispanics, and *PPP1R3B*/rs9987289 in African Blacks. The most significant SNPs for HDL-C levels were *KLF14*/rs4731702 in NHWs, *PPP1R3B*/rs9987289 in Hispanics, and *MMAB-MVK*/rs2338104 in African Blacks. The most significant SNPs for TG levels were *DOCK7*/rs10889353 in both NHWs and Hispanics, and *UBASH3B*/rs7941030 in African Blacks.

We were able to replicate the GWAS associations with the same lipid trait in the same direction in at least one ethnic group for 25 of 40 SNPs analyzed (40 in NHWs and Hispanics, 34 in African Blacks); at nominal significance (*p*<0.05) for 13 SNPs and with a trend for association for 12 SNPs. Five of 6 genome-wide significant TC loci, both of 2 genome-wide significant LDL-C loci, and 5 of 7 genome-wide significant TG loci were replicated in the same direction (at nominal significance or with a trend for association) in at least one ethnic group. Of 36 SNPs with genome-wide level significance and one with *p* = 7.7×10^−4^ for HDL-C levels, we were able to replicate the associations in the same direction for 18 SNPs in at least one ethnic group. Two SNPs showed significant but discordant results for association with HDL-C levels as compared to the original GWAS. For 12 SNPs, we observed significant associations with lipid traits other than those reported as genome-wide significant in the original GWAS, however we did not compare our results with GWAS findings that did not reach genome-wide level of significance. Because the nominal statistical significance (*p*<0.05) is considered acceptable in smaller follow-up studies on the established GWAS signals [19], we did not correct for multiple testing in our study. However, as stated above, we have also observed some new associations (i.e. significant associations with lipid traits other than those reported as genome-wide significant in the original GWAS), which should be considered ‘provisional’ until they are replicated in independent samples.

Overall the concordance rate was high for observed associations as compared to original GWAS findings (same allele with same effect or alternate allele with opposite effect); however, the associations were not consistently present in all ethnic groups that we studied. Other studies that investigated various ethnic groups also reported mixed results [11], [18], [20]–[22]. An independent replication study by Lanktree et al. [20] reported nominal association of the lead SNP or its proxy with the same lipid in the same direction for 13 of 32 GWAS loci tested in their modest-sized multiethnic sample (272 European, 330 South Asian, and 304 Chinese subjects from Canada). Another replication study by Keebler et al. [21] used a larger multiethnic sample (1,627 non-Hispanic Blacks, 1,659 Mexican Americans, and 2,230 NHWs from the US) and found mixed evidence by ethnic group for 17 lipid loci (19 successfully genotyped SNPs) examined, except for five loci (most of which established) that were shared by all groups. The largest GWAS by Teslovich et al. (a meta- analysis of 46 lipid GWAS comprising >100,000 individuals of European descent) [11] also sought replication in European (n = 7,000) and non-European samples (including >8,000 African Americans) and was able to replicate most, but not all, of the genome-wide significant signals identified in their primary analysis. The number of replicated SNPs was 35 of 36 for LDL-C, 44 of 47 for HDL-C, and 29 of 32 for TG in European replication sample while the replication rate was lower in African American replication sample (LDL-C: 33 of 36, HDL-C: 37 of 44 and TG: 24 of 30). In addition, for some loci, the observed effects were discordant in their replication samples. A recent multiethnic replication study by Chang et al. [22] examined 57 SNPs (55 GWAS-identified) in 3 major racial/ethnic groups from the US (2,296 NHWs, 1,699 non-Hispanic Blacks and 1,713 Mexican Americans) and replicated less than 67%, 44%, and 44% of GWAS-validated SNPs in each ethnic group, respectively. Another recent multiethnic replication study by Dumitrescu et al. [18] investigated 49 GWAS-identified SNPs in six racial/ethnic groups that included larger samples of European Americans (∼20,000), African Americans (∼9,000) and Mexican Americans/Hispanics (∼2,500). Based on nominal significance and consistent direction of effect, they were able to replicate the majority of associations with HDL-C, LDL-C, and TG in European Americans (85%, 95%, and 100%, respectively) and generalize a number of them to African Americans (44%, 58%, and 57%) and Mexican Americans/Hispanics (52%, 53%, and 86%). Overall, the effect sizes they observed in European Americans were smaller than previously reported estimates and, despite their adequately powered study, they failed to replicate some loci even in European Americans, which was predominantly the case for HDL-C loci (15% of SNPs failed to replicate) than LDL-C (5%) and TG (0%) loci.

Although our study has focused only on newly identified GWAS signals and did not include established genes with well-known functions in lipoprotein metabolism (which were most consistently replicated across various studies and/or various ethnic groups), our observations were similar to those in other multiethnic studies in terms of not being able to replicate all associations, observing lower replication rates in non-European populations (Table 4), and obtaining lower replication rates for HDL-C levels (page 12). Although our samples sizes were smaller as compared to two recent multiethnic replication studies by Chang et al. [22] and Dumitrescu et al. [18], our study examined several additional GWAS-identified variants that were not included in those studies.

Gender-specific effects on the genetics of lipid traits have also been suggested. We used gender as a covariate in our analyses but did not perform gender-stratified analyses due to power-related concerns. When Teslovich et al. [11] reanalyzed their GWAS data separately in men and women, they observed gender-specific effect sizes or gender-specific associations at some (but not many) loci.

In conclusion, our study shows evidence of replication for several, but not all, genome-wide significant SNP associations in our multiethnic sample. Lack of replication of genetic associations or the observation of different associations (with different lipid traits) may be related to a number of factors, such as insufficient power, differences in study population characteristics, allelic/genetic heterogeneity, allele frequency and/or linkage disequilibrium (LD) differences across various ethnic groups, population stratification, differences in effect sizes, and gene-environment interactions. Replication of some loci with small effect sizes (captured after meta-analysis of several GWAS by including several thousand subjects) may require the accumulation and meta-analysis of several independent replication studies (such as this study). The difficulty of generalizing the associations to various racial/ethnic populations may also be related to the fact that most GWAS-identified variants are likely to be in LD with the functional variant(s) rather than being functional themselves and LD patterns often vary across populations, which would affect the strength of indirect associations. In fact, a recent large study by Musunuru et al. [23] that performed dense genotyping in various lipid loci in a large sample set comprising of European Americans and African Americans, reported that many loci showed major differences in genetic architecture between these two ethnic groups and the most significant SNP at a given locus for a given trait often varied among them.

Further characterization of relevant lipid loci is necessary through their comprehensive sequencing in individuals with extreme phenotypes followed by functional evaluation of identified variants. Among several loci identified to date, the top priority could be given to those found to be relevant to more than one lipid trait and/or confirmed in more than one ethnic group.
